# Numerical Laminate Screening of Pin-Loaded CFRP Driveshaft Joints Using Progressive Damage Analysis and Multi-Criteria Evaluation

**DOI:** 10.3390/polym18192435

**Published:** 2026-10-07

**Authors:** Liming Zheng, Lingze Yuan, Nengwen Wang, Xintian Liu

**Affiliations:** 1 College of New Energy Automotive Engineering, Luoyang Vocational College of Science and Technology, Luoyang 471822, China; zhengliming123@lku.edu.cn; 2 School of Mechanical and Automotive Engineering, Shanghai University of Engineering Science, Shanghai 201620, China; lingze202409@163.com (L.Y.); nengwen1228@163.com (N.W.)

**Keywords:** CFRP driveshaft joint, laminate architecture, progressive damage, failure mechanism, multi-criteria evaluation

## Abstract

Carbon fiber-reinforced polymer (CFRP) driveshafts offer significant lightweight potential, but the performance of pin-loaded joints is strongly affected by laminate architecture. To support laminate selection beyond peak strength alone, a numerical screening framework combining finite-element analysis, Hashin-based progressive damage modeling, and multi-criteria evaluation was developed. Seven representative layups were compared under axial compression considering off-axis fiber orientation, stacking order, hoop restraint, and mid-plane symmetry. The results showed that Layup 3 achieved the highest peak load of 28.38 kN but exhibited abrupt brittle failure, whereas Layup 1 reached 22.02 kN with more gradual post-peak degradation and localized progressive damage. Removal of hoop-oriented plies promoted longitudinal splitting-type deformation, while asymmetric stacking reduced deformation stability. By integrating peak load, effective energy absorption, and failure safety, Layup 1 achieved the highest comprehensive evaluation index of 0.612 and remained preferred when the safety weight exceeded approximately 0.2. Within the present numerical framework, the proposed approach provides a practical basis for preliminary laminate screening of CFRP driveshaft joints.

## 1. Introduction

Lightweight transmission components have received increasing attention because of the demands for lower rotating mass, higher efficiency, and improved dynamic performance. CFRP driveshafts have therefore been investigated through structural optimization, vibration analysis, and finite-element assessment, with laminate design identified as a key factor affecting stiffness and load transfer [1,2,3]. Composite–metal connections and damage monitoring have also attracted attention because local joint regions may limit the structural advantages of CFRP shafts [4,5]. Temperature-dependent fatigue studies further indicate that load-carrying performance and service reliability should be considered together [6].

High stress gradients are generated around mechanically fastened composite joints, where fiber damage, matrix cracking, splitting, and net-section failure may occur. Progressive-damage models have been widely applied to reproduce nonlinear load transfer and failure evolution in CFRP joints [7,8,9]. Recent studies have further incorporated tightening processes, fastener geometry, and stiffness degradation to improve the prediction of local bearing damage [10,11]. Residual stress and crack propagation have also been shown to affect the durability of composite automotive structures [12]. These findings highlight the need to evaluate damage evolution rather than relying solely on ultimate strength.

Laminate architecture further modifies the mechanical response of composite joints. Stacking sequence and fiber orientation have been shown to alter load distribution, deformation, damage propagation, and buckling characteristics in mechanically connected laminates and composite tubes [13,14,15]. Fatigue damage and residual strength have also exhibited pronounced dependence on stacking sequence [16], while residual stress-sensitive life behavior has been observed in automotive mechanical components [17]. A laminate dominated by a single reinforcement direction may therefore provide high strength in one response mode while remaining vulnerable to unfavorable deformation or localized failure.

Effects of frictional load transfer, cyclic loading, joint geometry, and assembly conditions have also been reported for composite joints [18,19,20,21], while random-load fatigue behavior remains sensitive to crack-growth characteristics in automotive composite structures [22]. Experimental and numerical investigations have further characterized fatigue damage evolution in mechanically fastened CFRP joints [23,24]. These findings indicate that stable damage development and sufficient deformation tolerance should be considered during the initial structural design stage.

Recent research has focused on damage-aware joint design. Hole-making quality, installation damage, and thin-ply bearing behavior have been examined experimentally and numerically [25,26,27], together with alternative fastening schemes and laminate systems [28,29]. Nonlinear damage accumulation and temperature effects have also been considered [30,31,32,33]. Reliability-oriented assembly, multiscale modeling, preload uncertainty, installation effects, and local reinforcement have been reported during 2025–2026 [34,35,36,37,38], while continuum-damage and forced-assembly models have extended joint-failure prediction under different loading conditions [39,40].

Recent studies have also highlighted the influence of manufacturing conditions and loading rate on the anisotropic response of fiber-reinforced polymer composites. Zhang et al. showed that process parameters and the associated porosity can significantly affect the longitudinal, transverse, and interlayer mechanical properties of short-carbon fiber-reinforced polymer composites [41]. Yang et al. further demonstrated that the strength, stiffness, and damage evolution of CFRP composites can exhibit pronounced strain-rate dependence under dynamic loading [42].

Laminate architecture strongly influences load transfer and deformation in CFRP joints. Longitudinal plies mainly provide axial stiffness, off-axis plies assist shear transfer and stress redistribution, and hoop-oriented plies restrain circumferential deformation. Fiber orientation and stacking sequence therefore affect local stiffness and damage development around the joint region [13]. Pin–hole contact further introduces concentrated bearing and shear stresses, leading to competing failure modes [20,21].

However, CFRP driveshaft design and joint-failure analysis are still often treated separately. Existing studies mainly address global driveshaft performance or joint behavior for prescribed laminates, while the combined effects of off-axis angle, stacking order, hoop restraint, and laminate symmetry remain insufficiently investigated.

To address this gap, seven representative layups were compared within a unified numerical screening framework. Progressive damage analysis was combined with peak load, effective energy absorption, and failure safety to distinguish high-strength brittle responses from more balanced progressive-failure behavior.

## 2. Materials and Methods

### 2.1. Laminate Mechanics and Design Rationale

The mechanical response of CFRP driveshaft joints is governed by ply orientation and stacking sequence. Longitudinal plies mainly provide axial stiffness, off-axis plies contribute to shear resistance and load redistribution, and hoop-oriented plies restrain circumferential deformation. A balanced laminate architecture should therefore coordinate these load-carrying functions while limiting undesirable coupling, providing the basis for the layup configurations examined in the subsequent numerical analysis.

The stiffness contribution of each ply changes with its orientation relative to the longitudinal axis of the driveshaft. For an orthotropic lamina oriented at an angle *θ*, The transformed stiffness matrix can be given as follows:

(1) $$\bar{Q}=T^{-1}QT^{-T}$$

where **Q** denotes the reduced stiffness matrix in the material coordinate system, $\bar{Q}$ is the transformed stiffness matrix in the structural coordinate system, and **T** is the transformation matrix associated with the ply angle. The transformed stiffness components vary nonlinearly with *θ*, allowing the contribution of different fiber orientations to the global axial and shear response to be evaluated directly.

**Figure 1:**
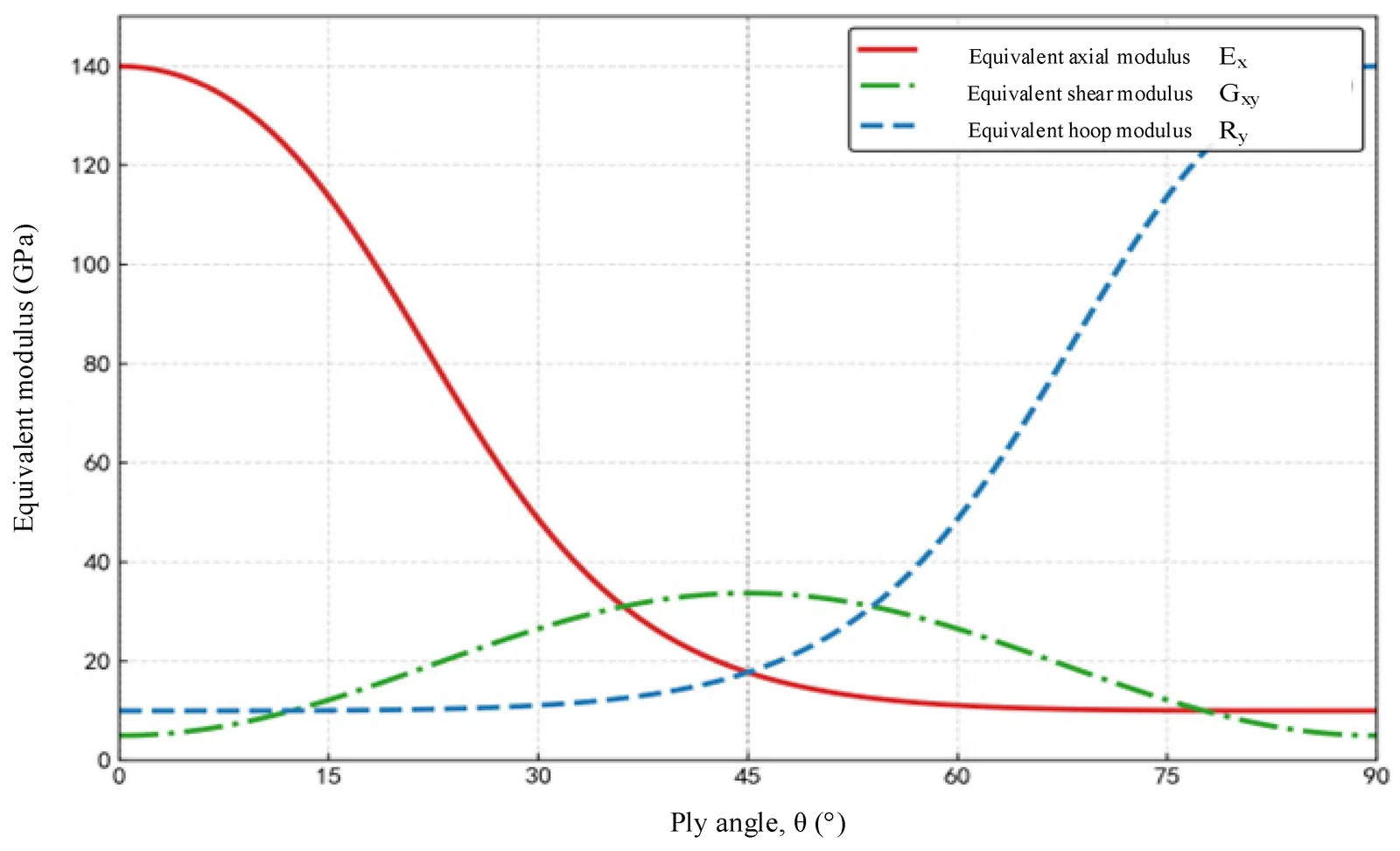
Variation in equivalent moduli with ply orientation.

As shown in Figure 1, axial stiffness decreased with increasing ply angle, while the in-plane shear stiffness reached a high level near ±45°. The 0° plies mainly supported axial loading, ±45° plies contributed to shear resistance and stress redistribution, and 90° plies provided circumferential restraint around the joint region. A mixed layup was therefore required to balance axial, shear, and hoop responses.

Stacking sequence can markedly affect the bearing response and load-carrying capacity of pin-loaded composite laminates [43]. The laminate force–moment relation is given as follows:

(2) $$\left[\begin{array}{c} N\\ M \end{array}\right]=\left[\begin{array}{cc} A & B\\ B & D \end{array}\right]\left[\begin{array}{c} \varepsilon^{0}\\ \kappa \end{array}\right]$$

where $N={\left[N_{x},N_{y},N_{\mathrm{xy}}\right]}^{T}$ and $M={\left[M_{x},M_{y},M_{\mathrm{xy}}\right]}^{T}$ denote the in-plane force resultants and moment resultants per unit width, respectively. $\varepsilon^{0}={\left[\varepsilon_{x}^{0},\varepsilon_{y}^{0},\gamma_{\mathrm{xy}}^{0}\right]}^{T}$ represents the mid-plane strain vector, and $\kappa ={\left[\kappa_{x},\kappa_{y},\kappa_{\mathrm{xy}}\right]}^{T}$ is the laminate curvature vector. The matrices **A**, **B**, and **D** denote the extensional stiffness, extension–bending coupling stiffness, and bending stiffness matrices, respectively. For a laminate symmetric about the mid-plane, the coupling matrix **B** vanishes, thereby suppressing extension–bending coupling. For the nominal axial loading condition considered here, no external moment resultant is prescribed. The moment equilibrium derived from Equation (2) can therefore be expressed as follows:

**B*ε***^0^ + **D*κ*** = 0 (3)

***κ*** = −**D**^−1^**B*ε***^0^ (4)

This relation shows that a non-zero coupling matrix in an asymmetric laminate can convert in-plane deformation into additional bending curvature, whereas this coupling response is suppressed for a symmetric laminate. In addition, a balanced off-axis arrangement reduces the extension–shear coupling associated with *A*_16_ and *A*_26_. Therefore, laminate symmetry and balance were treated as separate design factors because they influence different coupling mechanisms and deformation modes.

To characterize the influence of off-axis fiber orientation, 45° was selected as the baseline angle because it provides a high in-plane shear contribution. The 30° and 60° orientations were introduced to represent two contrasting stiffness tendencies: 30° retains a greater axial contribution, whereas 60° increases the relative transverse and circumferential contribution while reducing axial stiffness. These three angles therefore provide a controlled comparison of the transition from axial-dominated to off-axis-dominated behavior. In addition to fiber orientation, stacking order, hoop restraint, and mid-plane symmetry were treated as independent design variables because they directly affect stiffness coupling and deformation stability.

For the ±45°-only limiting laminate, the intrinsic tendency for circumferential expansion was further quantified using the laminate extensional stiffness. Under idealized axial membrane loading with no externally applied circumferential force resultant,

(5) $$N_{\theta }=A_{12}\varepsilon_{x}^{0}+A_{22}\varepsilon_{\theta }^{0}=0$$

A pre-damage transverse-expansion ratio can therefore be defined as follows:

(6) $$R_{\mathrm{\theta x}}=\left|\frac{\varepsilon_{\theta }^{0}}{\varepsilon_{x}^{0}}\right|=\frac{A_{12}}{A_{22}}$$

Using the material properties listed in Table 1 for the ${\left[\pm 45^{\circ }\right]}_{4}$ laminate gives *R_θx_* = 0.765. This value represents an idealized linear elastic membrane estimate before substantial damage and geometric nonlinearity develop and is used to quantify the intrinsic circumferential-expansion tendency rather than the post-damage ovalization magnitude.

### 2.2. Finite Element Model and Progressive Damage

**Figure 2:**
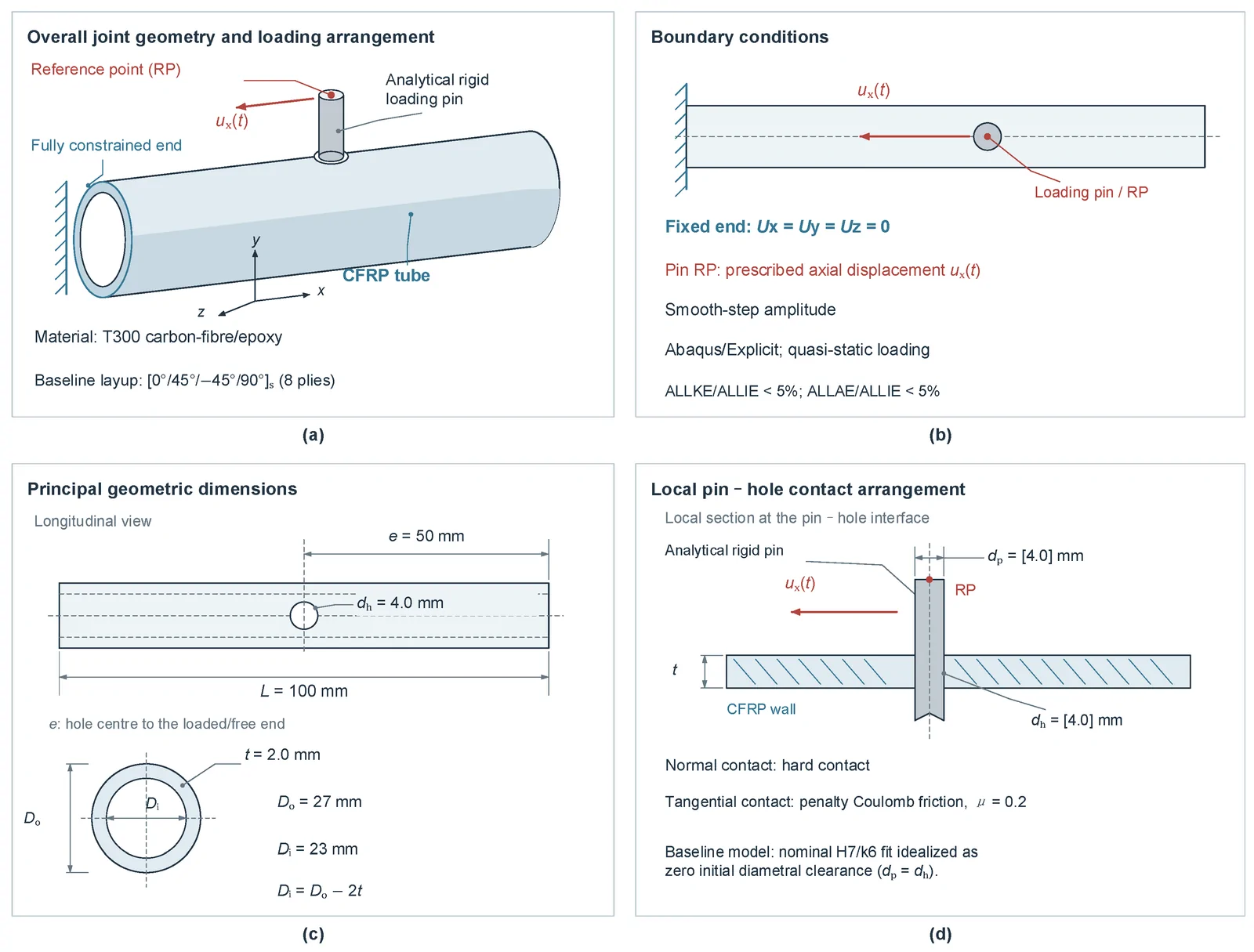
Schematic of the joint model: (**a**) overall joint geometry and loading arrangement; (**b**) boundary conditions; (**c**) principal geometric dimensions; (**d**) local pin–hole contact arrangement.

A three-dimensional numerical model comprising the CFRP tube and loading pin was established using Abaqus/Explicit 2022 (Dassault Systèmes Simulia Corp., Johnston, RI, USA). An explicit solution scheme was adopted to accommodate contact nonlinearity, local deformation, and progressive stiffness degradation during the joint response. The overall joint geometry, principal dimensions, boundary conditions, and loading arrangement are summarized in Figure 2. The CFRP tube had an overall length of *L* = 100 mm, a mid-surface diameter of 25 mm, and a total laminate thickness of *t* = 2.0 mm. With the shell reference surface located at the laminate mid-plane, the corresponding outer and inner diameters were *D_o_* = 27 mm and *D_i_* = 23 mm, respectively. The diametrically opposed pin holes had a diameter of *d_h_* = 4.0 mm and were centered 50 mm from the tube end. Under the nominal zero-clearance idealization, the corresponding pin diameter was *d_p_* = *d_h_* = 4.0 mm.

The nominal pin–hole fit was specified as H7/k6, which represents a transition fit and may result in a small clearance or interference depending on the actual dimensional tolerances. Because the objective of the present analysis was to compare laminate architectures rather than to reproduce a specific assembly tolerance, zero diametral clearance was adopted as the baseline condition. This neutral idealization avoids both the initial free motion associated with positive clearance and the assembly induced contact pressure associated with interference, thereby providing a common initial contact state for all laminate configurations. The zero-clearance condition should therefore be regarded as a nominal screening assumption rather than an exact representation of every H7/k6 assembly. No independent sensitivity analysis with respect to clearance or interference was available in the present numerical dataset; therefore, the effect of pin–hole fit tolerance is treated as a limitation of the present screening framework.

The CFRP tube was discretized using eight-node linear hexahedral reduced-integration elements (C3D8R). Local mesh refinement was applied around the pin hole to capture the high stress gradient generated by localized loading. A three-level mesh-sensitivity study was conducted using the baseline Layup 1 while keeping the material properties, boundary conditions, loading procedure, and damage parameters unchanged. The coarse, medium, and fine meshes employed hole-edge element sizes of 0.75, 0.50, and 0.35 mm, respectively, with corresponding far-field element sizes of 3.0, 2.0, and 1.4 mm. Peak load *P*_max_ and effective energy absorption *EA_eff_* were selected as the convergence indicators because both quantities are used in the subsequent multi-criteria evaluation. The quantitative mesh-sensitivity results are presented in Section 3.1. Based on this comparison, the medium mesh with a 0.50 mm hole-edge size and a 2.0 mm far-field size was adopted for the subsequent laminate-screening analyses. Each ply was independently discretized through the thickness to retain the layer-dependent stress and damage response. Enhanced stiffness-based hourglass control was adopted for the reduced-integration elements, and the artificial strain energy (ALLAE) was maintained below 5% of the internal energy (ALLIE) during the calculation.

The laminate was modeled using a T300/5113 carbon-fiber/epoxy property set reported in Ref. [44]. The complete orthotropic properties employed in the finite-element model are summarized in Table 1. These properties were used consistently for all seven laminate configurations and were treated as nominal material parameters rather than batch-specific measurements of manufactured specimens.

Progressive damage formulations have been widely employed to characterize damage initiation, stiffness degradation, and failure evolution in mechanically fastened composite laminates [45]. Progressive intralaminar damage was described using the Hashin failure criterion coupled with a continuum damage mechanics formulation. The effective stress state can be obtained as follows:

(7) $$\left[\begin{array}{c} {\hat{\sigma }}_{11}\\ {\hat{\sigma }}_{22}\\ {\hat{\tau }}_{12} \end{array}\right]=\left[\begin{array}{ccc} \frac{1}{1-d_{f}} & 0 & 0\\ 0 & \frac{1}{1-d_{m}} & 0\\ 0 & 0 & \frac{1}{1-d_{s}} \end{array}\right]\left[\begin{array}{c} \sigma_{11}\\ \sigma_{22}\\ \tau_{12} \end{array}\right]$$

where *d_f_*, *d_m_*, and *d_s_* denote the fiber, matrix, and in-plane shear damage variables, respectively. Four competing intralaminar failure modes were evaluated in the effective stress space: fiber tension, fiber compression, matrix tension, and matrix compression.

For fiber tensile loading $\left({\hat{\sigma }}_{11}\geq 0\right)$, the failure index is given as:

(8) $$F_{f}^{T}={\left(\frac{{\hat{\sigma }}_{11}}{X_{T}}\right)}^{2}+{\left(\frac{{\hat{\tau }}_{12}}{S_{L}}\right)}^{2}\geq 1$$

For fiber compression $\left({\hat{\sigma }}_{11}<0\right)$, the failure index is given as follows:

(9) $$F_{f}^{C}={\left(\frac{{\hat{\sigma }}_{11}}{X^{C}}\right)}^{2}\geq 1$$

For matrix tension $\left({\hat{\sigma }}_{22}\geq 0\right)$, the failure index is given as follows:

(10) $$F_{m}^{T}={\left(\frac{{\hat{\sigma }}_{22}}{Y_{T}}\right)}^{2}+{\left(\frac{{\hat{\tau }}_{12}}{S_{L}}\right)}^{2}\geq 1$$

For matrix compression $\left({\hat{\sigma }}_{22}<0\right)$, the failure index is given as follows:

(11) $$F_{m}^{C}={\left(\frac{{\hat{\sigma }}_{22}}{2S_{T}}\right)}^{2}+\left[{\left(\frac{Y_{C}}{2S_{T}}\right)}^{2}-1\right]\frac{{\hat{\sigma }}_{22}}{Y_{C}}+{\left(\frac{{\hat{\tau }}_{12}}{S_{L}}\right)}^{2}\geq 1$$

where *X_T_* and *X_C_* are the longitudinal tensile and compressive strengths, *Y_T_* and *Y_C_* denote the transverse tensile and compressive strengths, and *S_L_* and *S_T_* are the longitudinal and transverse shear strengths. Damage initiation occurs when the corresponding Hashin failure index reaches unity. After initiation, the affected material point enters an energy-based progressive softening stage rather than losing its load-carrying capacity instantaneously. The four mode-dependent critical fracture energies used for damage evolution are summarized in Table 2, corresponding to fiber tension, fiber compression, matrix tension, and matrix compression.

To reduce mesh-size sensitivity during strain softening, the damage evolution is regularized in terms of equivalent displacement using the characteristic element length *L_c_*,

*δ_eq_*_,*I*_ = *L_c_ε_eq_*_,*I*_ (12)

where *I* ∈ {*ft*,*fc*,*mt*,*mc*} denotes fiber tension, fiber compression, matrix tension, and matrix compression, respectively. The mode-dependent fracture energy governs the energy dissipated during the evolution from damage initiation to complete loss of load-carrying capacity. Fiber and matrix degradation are determined from their corresponding tensile and compressive damage variables, while the in-plane shear degradation incorporates the interaction among all four intralaminar failure modes.

The in-plane shear damage is given as:

*d_s_* = 1 − (1 − *d_ft_*)(1 − *d_fc_*)(1 − *d_mt_*)(1 − *d_mc_*) (13)

where *d_ft_*, *d_fc_*, *d_mt_*, and *d_mc_* denote fiber-tension, fiber-compression, matrix-tension, and matrix-compression damage variables, respectively.

The resulting damage variables were introduced into the degraded constitutive matrix, allowing the stiffness to decrease progressively with the development of fiber and matrix damage. This formulation was used to describe the nonlinear response of the laminate during the preliminary comparison of different stacking configurations.

The present laminate-screening model accounts for intralaminar fiber and matrix damage only. No cohesive elements or cohesive surface interactions were defined between adjacent plies; therefore, interlaminar delamination initiation and propagation were not explicitly modeled. This simplification was adopted because the objective of the present model is the comparative screening of laminate architectures using global load response and intralaminar damage characteristics rather than a detailed prediction of ply interface crack growth. Consequently, splitting-type deformation observed in the numerical results is interpreted as a global intralaminar damage/deformation pattern and not as an explicit prediction of delamination propagation.

### 2.3. Boundary Conditions and Layup Configurations

The end of the CFRP tube away from the pin hole was fully constrained to represent the clamping condition. The pin-loaded boundary condition was represented through a rigid kinematic coupling at the hole region. The hole boundary was coupled to a reference point located at the pin-hole section, while the opposite tube end was coupled to the loading reference point. An axial displacement of 50 mm was applied through a smooth-step amplitude, producing the relative compressive loading required for the laminate-screening analysis.

The pin–hole contact condition can significantly affect the local mechanical response of mechanically fastened composite joints [46]. General contact was defined between the loading pin and the hole surface. Hard contact was adopted in the normal direction, allowing separation while preventing penetration. The tangential behavior was described using a penalty-based Coulomb friction model. A friction coefficient of *μ* = 0.2 was adopted as the nominal composite–metal contact condition in the baseline model. Because the actual friction coefficient can vary with surface condition, contact pressure, and assembly state, *μ* = 0.2 was treated as a modeling parameter rather than an exact material constant. The same pin-hole geometry, normal-contact formulation, and friction coefficient were used for all laminate configurations so that the effect of laminate architecture could be isolated. Because contact remained active during the prescribed displacement loading, compressive contact and frictional resistance could continue to contribute to the reaction force after substantial laminate damage had developed. To reduce abrupt inertial excitation in the explicit analysis, the prescribed displacement was applied using a Smooth Step amplitude. A fixed mass-scaling scheme was employed with a target stable time increment of 1.0 × 10^−7^ s for elements below this threshold. The kinetic-to-internal-energy ratio (ALLKE/ALLIE) was monitored throughout the analysis to assess whether the response remained quasi-static, while the artificial-to-internal-energy ratio (ALLAE/ALLIE) was monitored to evaluate numerical stability. Accordingly, the quasi-static interpretation was based on the resulting energy partition rather than on the use of mass scaling alone. Responses exhibiting a pronounced increase in ALLKE relative to ALLIE were not interpreted as strictly quasi-static.

The seven configurations were constructed as a controlled design matrix rather than selected independently. Layups 1–3 were used to examine the off-axis angle effect while retaining the same symmetric laminate form. Layup 4 changed the stacking order relative to the baseline to evaluate the influence of ply position. Layup 5 removed the 90° plies to isolate the role of hoop restraint, whereas Layup 6 introduced an asymmetric stacking sequence to examine extension–bending coupling. Layup 7 contained only ±45° plies and was used as a limiting case to evaluate the loss of the axial load-bearing skeleton. Together, these configurations provide representative contrasts among the principal laminate-design factors, as summarized in Table 3.

### 2.4. Multi-Criteria Performance Evaluation

The laminate configurations were evaluated using three mechanical indicators representing load capacity, energy dissipation, and failure safety. The peak load *P*_max_ was used to characterize the maximum load-carrying capacity. The effective energy absorption *EA* represented the energy dissipated during the effective load-bearing process, because the progressive-damage responses considered here do not exhibit a conventional material yield point, the displacement used for the failure-safety evaluation was defined operationally from the load–displacement curve. The pseudo-yield displacement, *δ_y_*, was defined as the displacement corresponding to the maximum load *P*_max_, i.e., the end of the initial load-rising stage. The ultimate failure displacement, *δ_u_*, was defined as the first post-peak displacement at which the load decreased to 20% of *P*_max_. The 20% threshold was applied consistently to all laminate configurations to distinguish the effective post-peak load-bearing response from the low-load residual contact and sliding response. It is used here as an operational numerical criterion rather than as a material-specific failure strain. The failure-safety index was therefore calculated as $\lambda =\frac{\delta_{u}}{\delta_{y}}$. A larger value of *λ* indicates a greater deformation reserve between the peak-load state and the loss of effective load-carrying capacity.

Because the three indicators have different dimensions and numerical ranges, min–max normalization was applied before comprehensive evaluation. The normalized value can be obtained as follows:

(14) $$N_{\mathrm{ij}}=\frac{X_{\mathrm{ij}}-X_{j}^{\min}}{X_{j}^{\max}-X_{j}^{\min}}$$

where *X_ij_* is the original value of the *j*th indicator for the *i*th laminate configuration, and $X_{j}^{\max}$ and $X_{j}^{\min}$ denote the corresponding maximum and minimum values among all configurations.

To distinguish effective structural energy absorption from post-failure frictional dissipation, the apparent energy absorption was defined as the total area under the load–displacement curve, whereas the effective energy absorption was limited to the effective load-bearing stage. For Layup 5, the transition displacement *δ_s_* was defined at the first abrupt post-peak load drop, marking the onset of macroscopic longitudinal splitting, as discussed in Section 3.2. Beyond *δ_s_*, the response was dominated by low-load contact and frictional sliding rather than effective load transfer. The apparent and effective energy absorptions were calculated as follows:

(15) $$EA_{\mathrm{app}}=\int_{0}^{\delta_{\mathrm{end}}}P(\delta )\mathrm{d\delta}$$

(16) $$EA_{\mathrm{eff}}=\int_{0}^{\delta_{s}}P(\delta )\mathrm{d\delta}=EA_{\mathrm{app}}-\int_{\delta_{s}}^{\delta_{\mathrm{end}}}P(\delta )\mathrm{d\delta}$$

where *P*(*δ*) is the axial load, *δ_end_* is the final displacement of the numerical loading history, and *δ_s_* denotes the transition from effective structural load transfer to the residual sliding stage. The interval [*δ_s_*,*δ_end_*] was excluded from *EA_eff_* because the corresponding work is predominantly associated with continued pin–hole contact and frictional sliding after the macroscopic splitting-type deformation has developed. Only *EA_eff_*, rather than *EA_app_*, was used in the subsequent normalization and CEI calculation. The CEI is given as follows:

(17) $$\mathrm{CE}I_{i}=\sum_{j=1}^{3}w_{j}N_{\mathrm{ij}}$$

where *w_j_* is the weight assigned to the *j*th indicator and $\overset{3}{\underset{j=1}{\sum }}w_{j}=1$. The weights *w_P_* = 0.35, *w_EA_* = 0.30, and *w_s_* = 0.35 were adopted as a reference weighting scenario rather than as a uniquely derived optimum. This reference case assigns comparable importance to load-carrying capacity and failure safety while retaining the contribution of energy absorption. To evaluate the dependence of the laminate selection on this prescribed preference, a continuous weight-sensitivity analysis was performed by varying the safety weight *w_s_*. The remaining weight, 1 − *w_s_*, was redistributed between peak load and effective energy absorption while preserving their reference ratio of 0.35:0.30 = 7:6, giving $w_{P}=\frac{7}{13}(1-w_{s})$, $w_{\mathrm{EA}}=\frac{6}{13}(1-w_{s})$.

Thus, the three weights satisfy *w_P_* + *w_EA_* + *w_s_* = 1 throughout the sensitivity analysis, and the reference combination 0.35/0.30/0.35 is recovered at *w_s_* = 0.35. This procedure allows the robustness of the laminate preference to be evaluated over a broader range of engineering priorities rather than relying on a single prescribed weighting combination.

## 3. Mechanical Response and Failure

### 3.1. Load-Carrying Response of Different Laminate Configurations

Before comparing the seven laminate configurations, the mesh sensitivity of the baseline Layup 1 was quantitatively evaluated, as summarized in Table 4. 

The peak load increased from 21.61 kN for the coarse mesh to 22.02 kN for the medium mesh and 22.10 kN for the fine mesh. The corresponding effective energy absorption increased from 42.80 to 44.10 and 44.50 J, respectively. Taking the fine mesh as the reference, the medium mesh differed by only 0.36% in peak load and 0.90% in effective energy absorption. In comparison, the coarse mesh showed differences of 2.22% and 3.82%, respectively. These results indicate that further refinement from the medium to the fine mesh produced only minor changes in the two response quantities used in the subsequent evaluation. Therefore, the medium mesh was retained as a compromise between numerical accuracy and computational cost.

**Figure 3:**
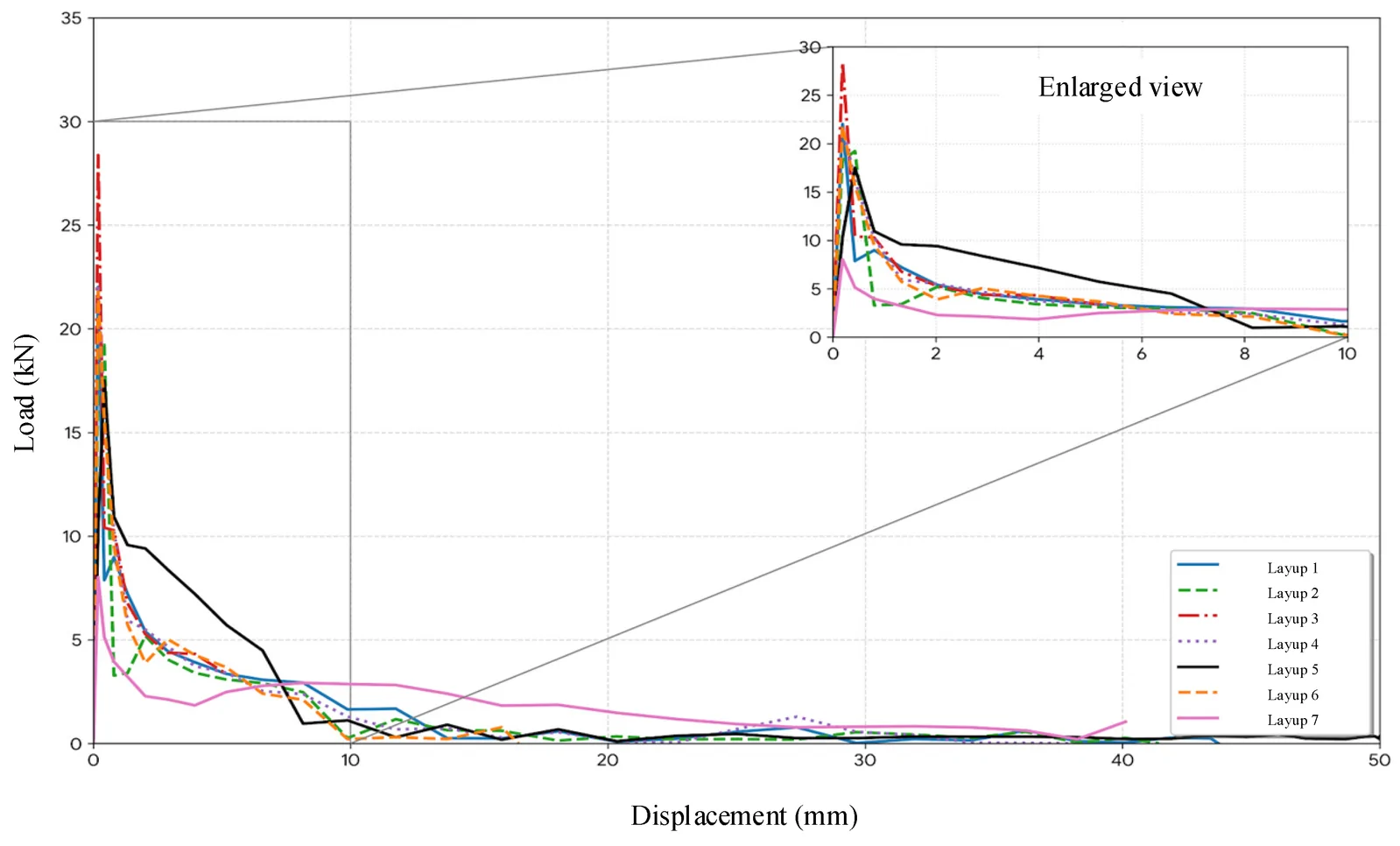
Load–displacement responses of different laminate configurations under axial compression.

The seven laminate configurations showed distinct axial-compression responses in stiffness, peak load, and post-peak behavior, exhibiting brittle-dominated, flexible, or progressive-damage characteristics. The load–displacement responses of the seven laminate configurations are shown in Figure 3.

Layup 3 exhibited the highest peak load of 28.38 kN together with a relatively high initial stiffness. After reaching the maximum load, the curve showed an abrupt reduction with almost no sustained post-peak load-carrying stage. This response was classified as brittle-dominated, indicating that a high peak load was accompanied by a rapid loss of structural capacity once the maximum load was exceeded.

Layup 7 showed a distinctly different response. Its peak load was only 8.02 kN, less than 40% of that of Layup 1, and the curve exhibited low stiffness together with a prolonged deformation stage. Layup 1 reached a peak load of 22.02 kN and maintained a more gradual reduction in load after the peak. The broader post-peak load-bearing region reflects a progressive response with greater deformation tolerance than the brittle-dominated configuration.

Several load–displacement curves converged to a similar secondary load level of approximately 3–4 kN after substantial post-peak degradation. This common residual level should not be interpreted as an identical residual material strength of the different laminates. After extensive intralaminar damage and stiffness degradation, the contribution of the intact laminate architecture to the global reaction force was markedly reduced, while the rigid pin remained in contact with the damaged hole surface under the prescribed displacement. The residual reaction was therefore increasingly governed by the remaining stiffness of the damaged laminate together with local pin–hole compression and frictional sliding. Because the pin geometry, normal hard-contact condition, and Coulomb friction coefficient (*μ* = 0.2) were identical for all configurations, these contact-controlled contributions became similar among several layups after severe damage, producing the comparable 3–4 kN secondary plateau. Accordingly, the secondary plateau represents a post-damage residual contact response rather than recovery of the primary laminate load-carrying capacity. This observation further demonstrates that peak load alone is insufficient to characterize the complete failure response of the investigated layups.

### 3.2. Layup-Dependent Failure Mechanisms

**Figure 4:**
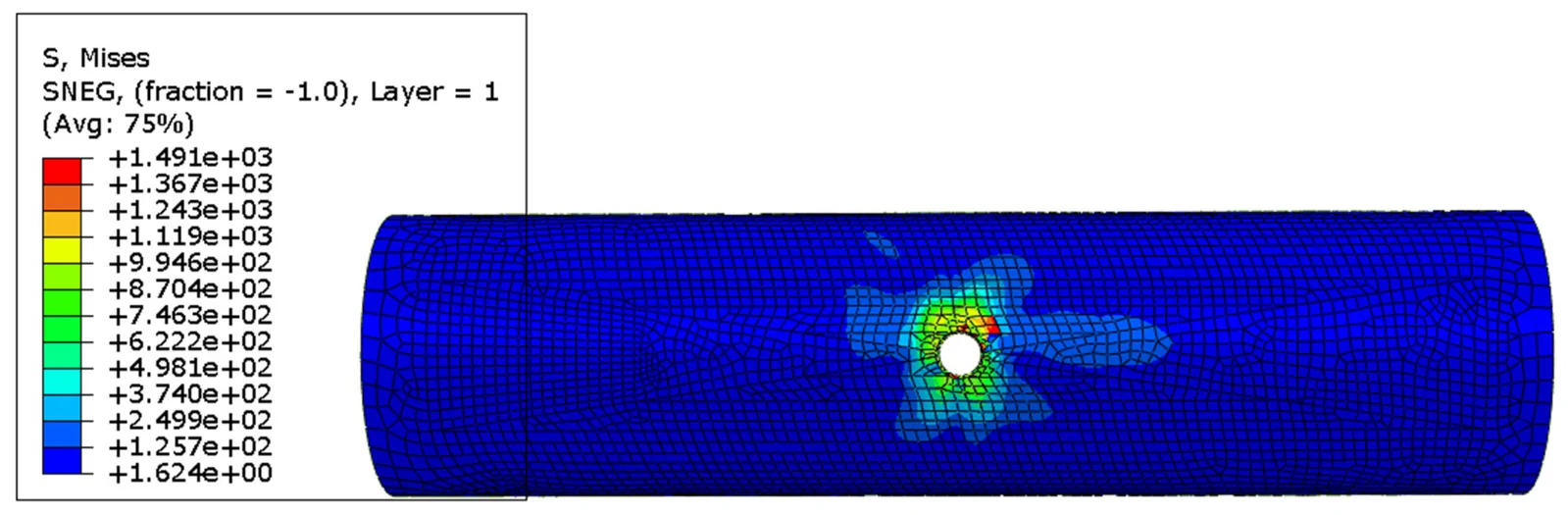
Von Mises stress distribution of Layup 1 near the pin hole.

The load-carrying response of pinned composite joints is closely associated with the local stress state around the loaded hole, which can lead to distinct bearing and net-tension failure modes [47]. The differences in load–displacement response were accompanied by distinct damage patterns among the laminate configurations. Representative numerical and schematic results were examined to identify the relationship between laminate architecture, local stress concentration, and dominant failure morphology. Figure 4 presents the quantitative von Mises equivalent-stress field from the original post-processing output, including the numerical contour legend and field-variable definition. In contrast, the subsequent schematic illustrations are used only to identify the spatial location and morphology of the dominant failure patterns. Accordingly, no quantitative comparison of damage magnitude is inferred from the colors or shaded regions in these qualitative illustrations. Particular attention was given to the baseline configuration, the high-angle configuration, the ±45°-dominated configuration, and the configuration without hoop-oriented plies.

**Figure 5:**
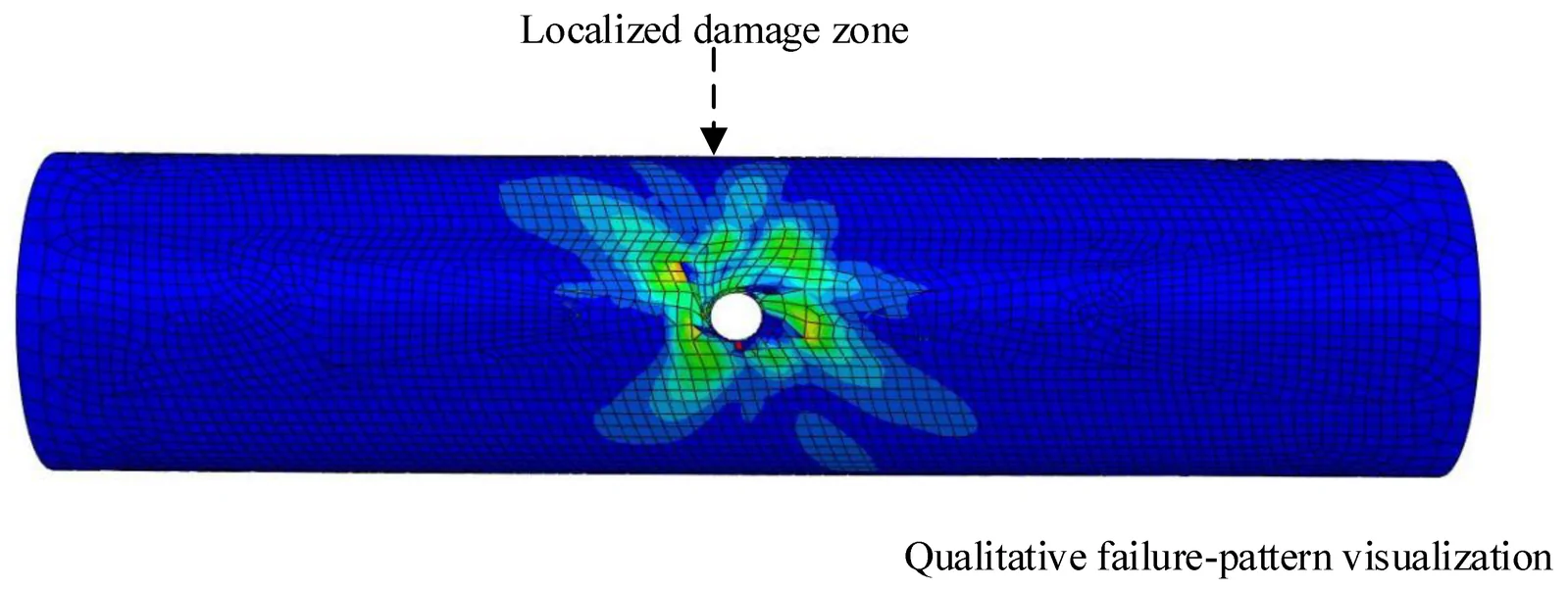
Localized failure pattern of Layup 1 at ultimate failure. Colors are used only for qualitative visualization and do not represent quantitative damage magnitudes.

Layup 1 exhibited a localized progressive damage pattern. The quantitative stress field in Figure 4 shows a pronounced concentration of von Mises equivalent stress around the pin–hole region. The highest stress region is localized adjacent to the loaded hole, consistent with the strong local stress gradient generated by pin loading. The von Mises field is reported only as a scalar indicator of local stress concentration; it is not used as a composite failure criterion. Intralaminar failure initiation and evolution are evaluated using the Hashin-based damage formulation described in Section 2.2. The qualitative ultimate-stage pattern shown in Figure 5 indicates that the affected region remained localized around the pin-hole bearing zone. No quantitative damage magnitude is inferred from the contour colors in Figure 5.

**Figure 6:**
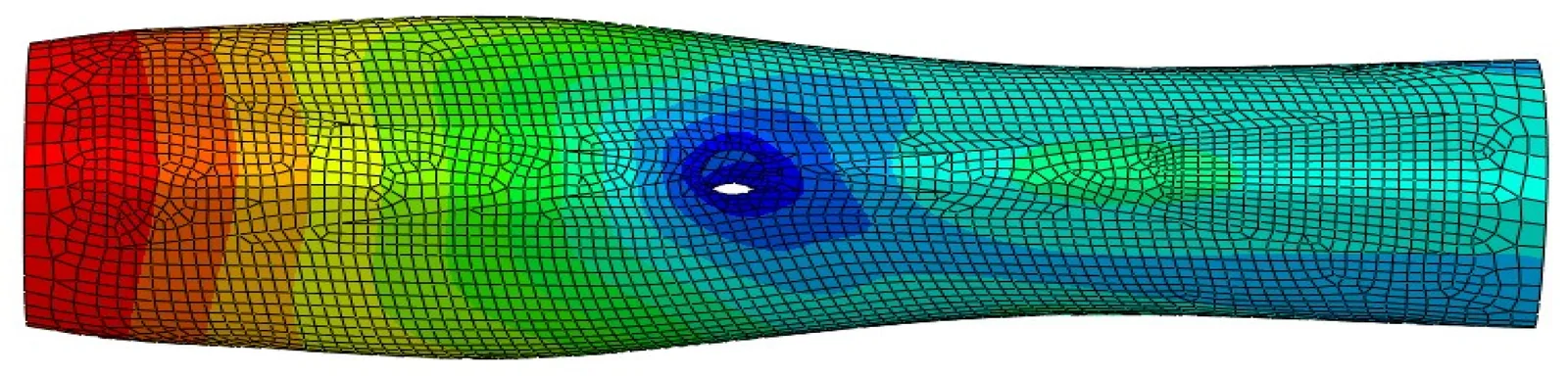
Large deformation of ±45° Layup 7 under axial compression. Colors are used only for qualitative visualization and do not represent quantitative field values.

A distinctly flexible deformation response was observed for Layup 7, which consisted only of ±45° plies. Its peak load was 8.02 kN, less than 40% of that of Layup 1, and its load–displacement response exhibited low stiffness together with an extended large-deformation stage. As defined in Section 2.1, the idealized pre-damage transverse-expansion ratio of this laminate is *R_θx_* = *A*_12_/*A*_22_ = 0.765, indicating a pronounced intrinsic tendency for axial deformation to be accompanied by circumferential deformation. The later-stage configuration shown in Figure 6 is qualitatively consistent with this tendency and exhibits nonuniform cross-sectional deformation. Because the contour image itself does not provide the nodal kinematic history required to determine a post-damage ovalization ratio, no quantitative ovalization magnitude is inferred from Figure 6. The absence of 0° plies reduces axial load-bearing stiffness, while the simultaneous absence of 90° plies weakens circumferential restraint.

**Figure 7:**
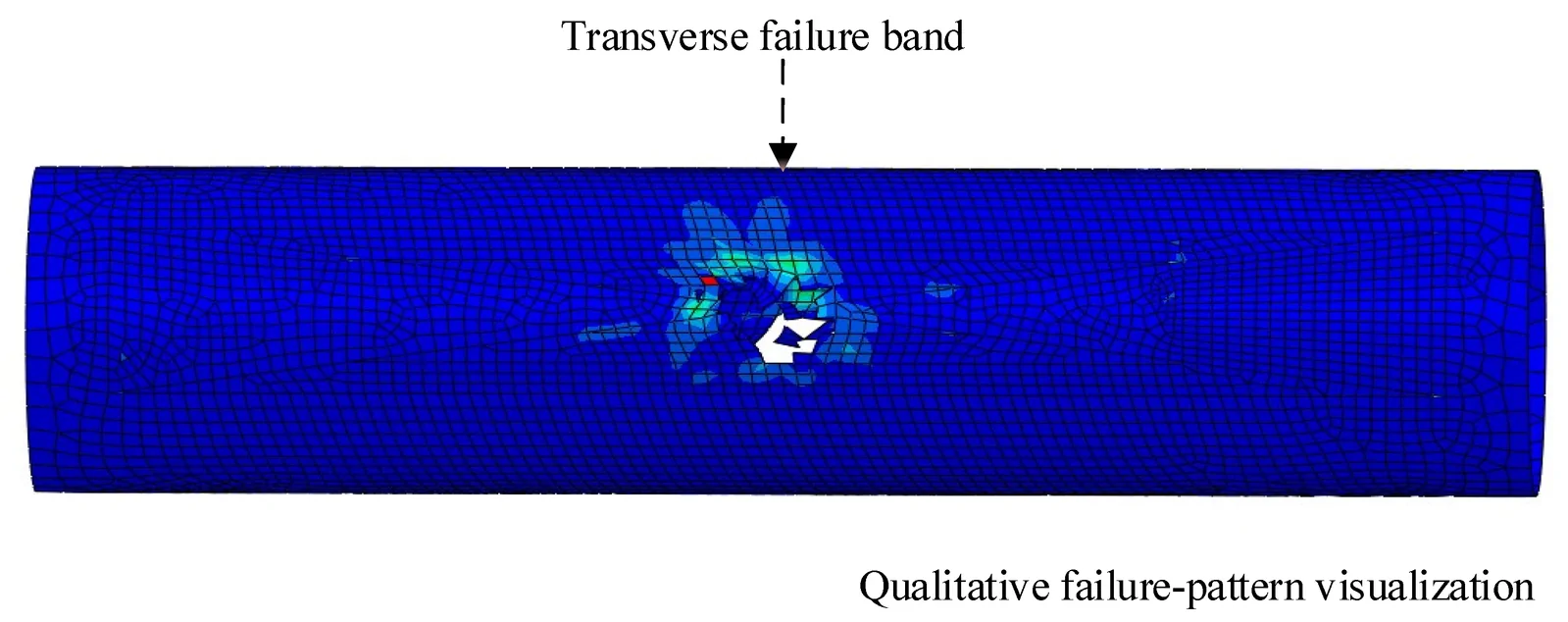
Transverse failure band and net-section failure of Layup 3. Colors are used only for qualitative visualization and do not represent quantitative damage magnitudes.

The transverse failure pattern of Layup 3 is illustrated in Figure 7.

**Figure 8:**
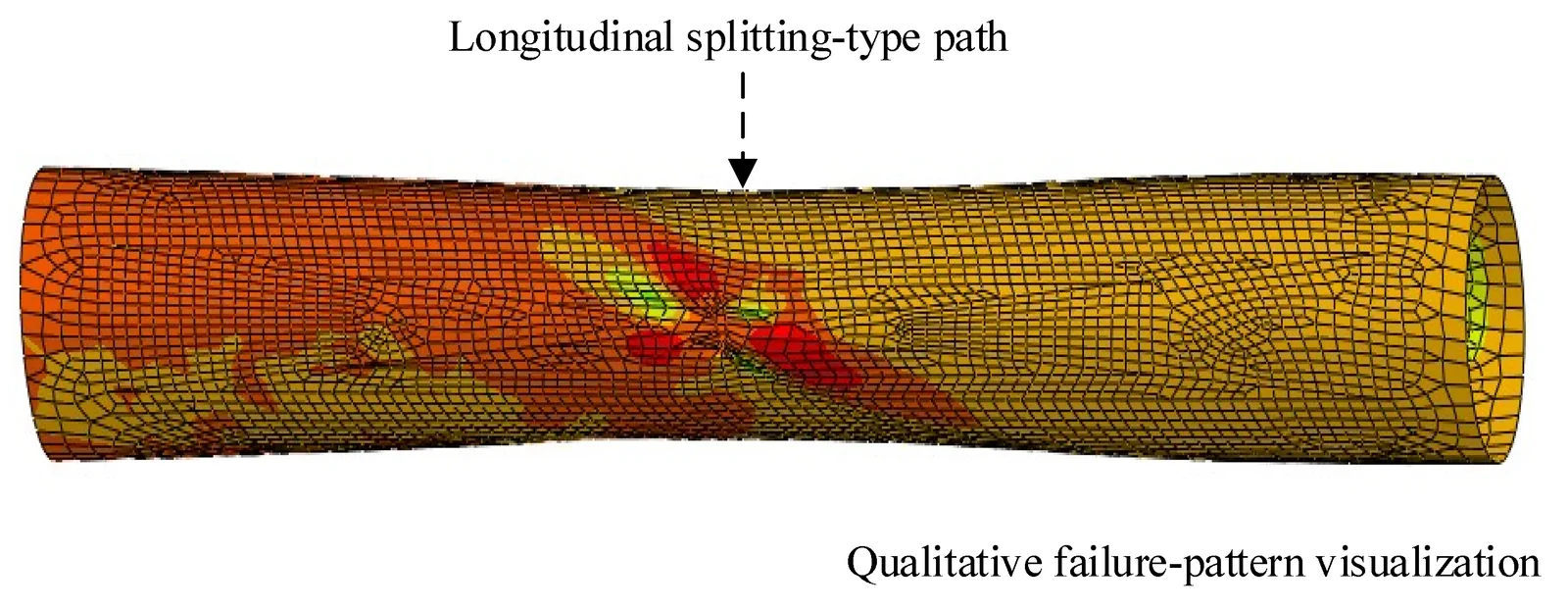
Longitudinal splitting of Layup 5 without hoop plies. Colors are used only for qualitative visualization and do not represent quantitative damage magnitudes.

Layup 3 showed a markedly more brittle failure pattern. Although the configuration reached the highest peak load, the damage field was characterized by transverse fracture across the load-bearing section. The high proportion of large-angle plies increased the circumferential constraint but reduced the contribution of fibers aligned with the loading direction. Once the local damage exceeded the load-bearing capacity of the remaining section, rapid net-section failure occurred, consistent with the characteristic failure modes reported for mechanically fastened composite laminates [48]. This failure mechanism corresponded to the abrupt post-peak load reduction observed in Figure 3. The longitudinal splitting-type response of Layup 5 is illustrated in Figure 8.

Removal of the hoop-oriented plies produced another distinct failure mode in Layup 5. A longitudinal splitting-type damage and deformation pattern developed near the pin-loaded region and extended along the axial direction as the surrounding laminate lost circumferential restraint. Because interlaminar cohesive interactions were not included in the present screening model, this pattern is interpreted as a global intralaminar damage/deformation mode rather than as an explicit simulation of delamination-crack initiation and propagation. Layup 5 exhibited an apparent energy absorption of 61.31 J when the entire load–displacement history was integrated. However, beyond the transition displacement *δ_s_*, the response entered a residual stage in which the remaining work was predominantly associated with continued contact and frictional sliding rather than effective structural load transfer. Therefore, the contribution from *δ_s_* to *δ_end_* was excluded according to the effective energy-absorption definition introduced in Section 2.4. The resulting response indicates the important role of hoop-oriented plies in suppressing longitudinal splitting-type deformation and maintaining the integrity of the joint region. Figure 8 is therefore interpreted as a schematic visualization of the longitudinal splitting-type path rather than as a quantitative damage-field contour.

### 3.3. Stacking Symmetry and Deformation Stability

The influence of stacking symmetry was examined by comparing the energy histories of the symmetric Layup 1 and the asymmetric Layup 6. Both configurations contained comparable principal ply orientations, while their stacking arrangements differed in mid-plane symmetry. The comparison was used to identify whether the coupling introduced by asymmetric stacking affected the deformation stability of the joint during explicit loading.

**Figure 9:**
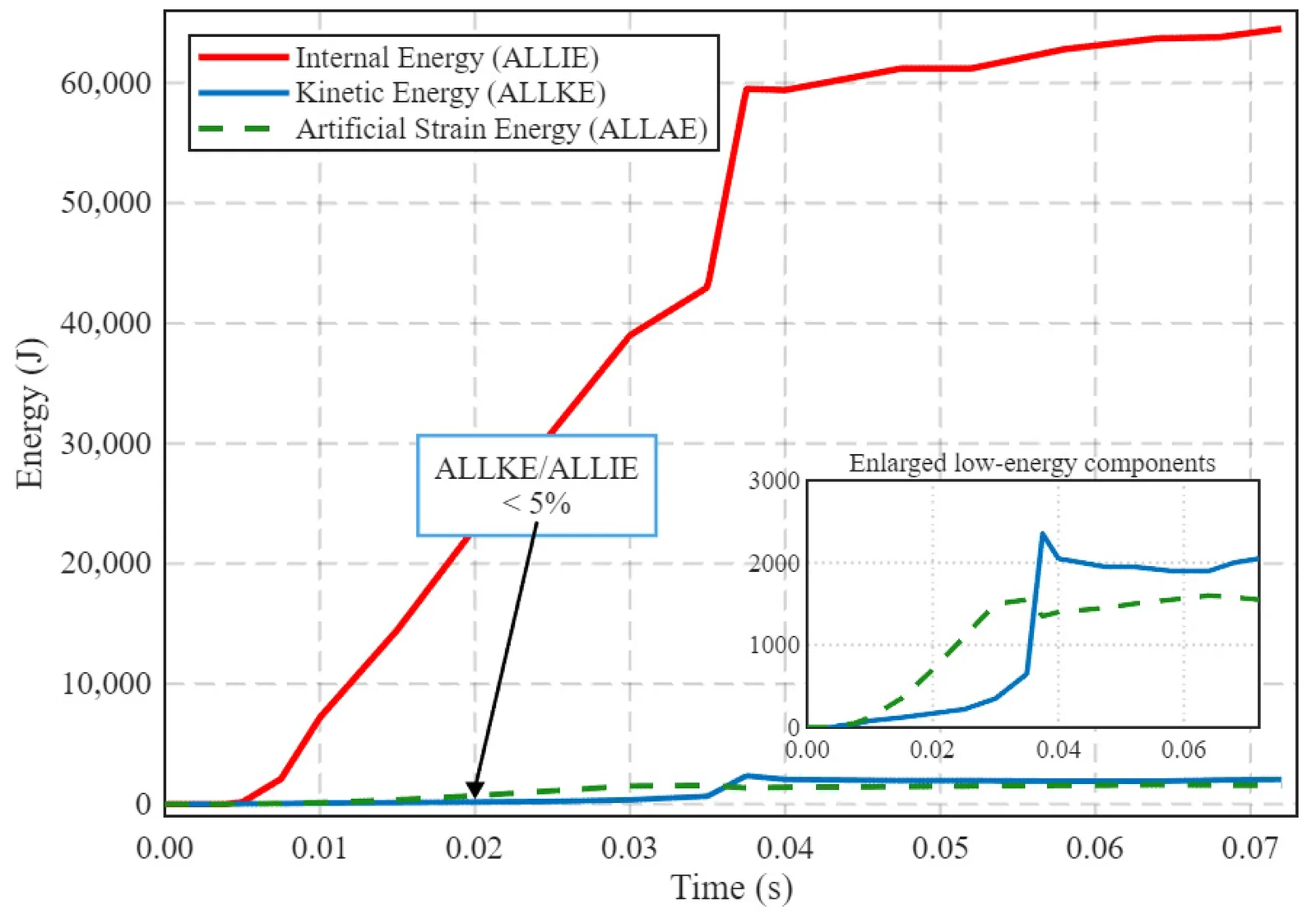
Energy evolution of Layup 1 during quasi-static loading.

Layup 1 exhibited a stable energy evolution throughout the loading process, as shown in Figure 9. The internal energy increased smoothly with increasing displacement and remained the dominant component of the system energy. The kinetic energy was maintained at a low level, with the ratio of kinetic energy to internal energy remaining below 5%. The artificial strain energy was also negligible compared with the internal energy. These characteristics indicate that the response of Layup 1 remained close to a quasi-static state without pronounced inertial disturbance during loading. This stable energy partition was therefore used as the reference quasi-static response for comparison with the asymmetric laminate under the same loading procedure.

**Figure 10:**
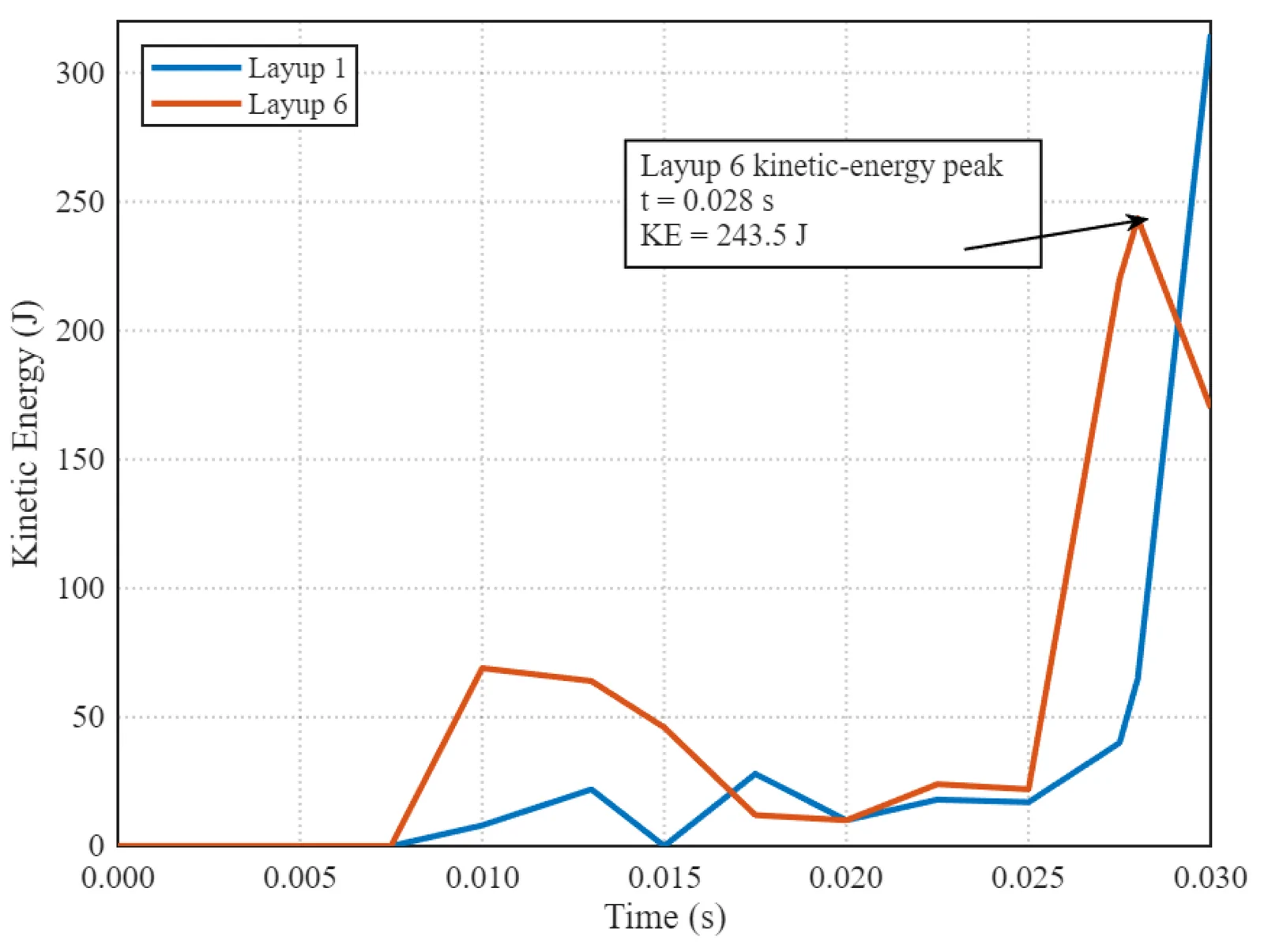
Kinetic-energy surge and transient instability of the asymmetric Layup 6.

A distinctly different energy response was observed for the asymmetric Layup 6. Its kinetic energy exhibited a step-like increase at approximately *t* = 0.01 s and subsequently reached a peak of 243.5 J at approximately *t* = 0.028 s. In contrast to Layup 1, for which ALLKE remained below 5% of ALLIE during the quasi-static portion of the response, the pronounced kinetic-energy increase in Layup 6 indicates that its response departed from the quasi-static regime. Because the explicit model employed fixed mass scaling and no independent loading-rate or mass-scaling sensitivity analysis is available for this configuration, the kinetic-energy surge cannot be attributed uniquely to a structural-instability mechanism. Accordingly, the post-surge response is not interpreted as a strictly quasi-static material response, and the kinetic-energy history alone is not used as evidence of a specific buckling or instability mode. The kinetic-energy surge of the asymmetric Layup 6 is shown in Figure 10.

The different energy responses are mechanically consistent with the extension–bending coupling introduced by the asymmetric stacking sequence. As derived from Equations (2)–(4), for the nominal axial loading condition with no prescribed external moment resultant, the laminate curvature ***κ*** is governed by Equation (4). For the symmetric Layup 1, **B** approaches zero, thereby suppressing the bending curvature induced by in-plane deformation. In contrast, the non-zero *B_ij_* terms of Layup 6 permit axial deformation to generate an additional bending response. The pronounced kinetic-energy increase in Layup 6 is therefore consistent with a greater tendency toward coupled bending deformation and reduced deformation stability. However, within the present numerical dataset, this observation is treated only as a comparative indicator and not as independent confirmation of a specific structural-instability or buckling mode.

### 3.4. Comprehensive Laminate Evaluation

**Figure 11:**
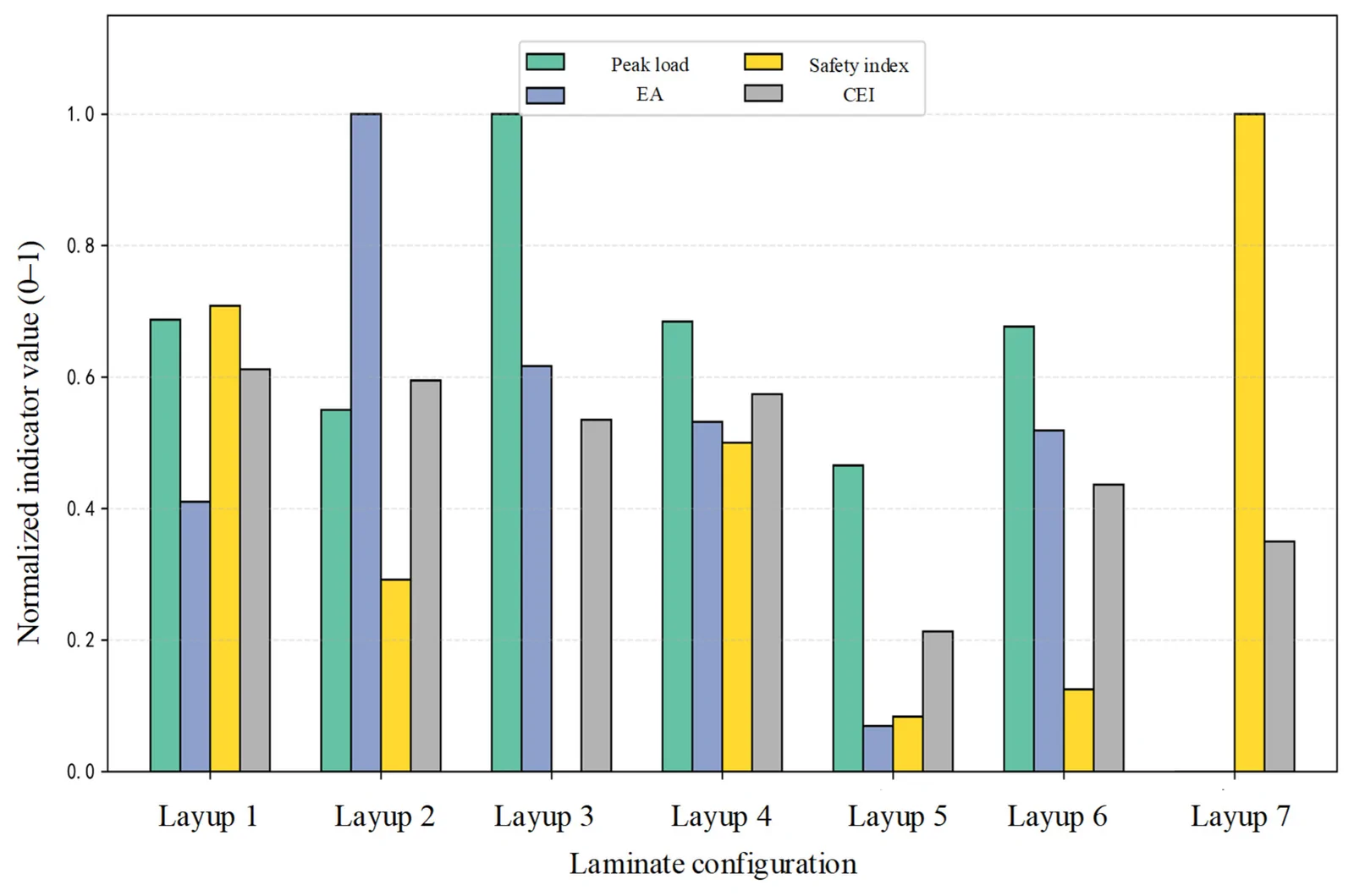
Normalized mechanical performance and comprehensive evaluation index of different laminate configurations.

**Figure 12:**
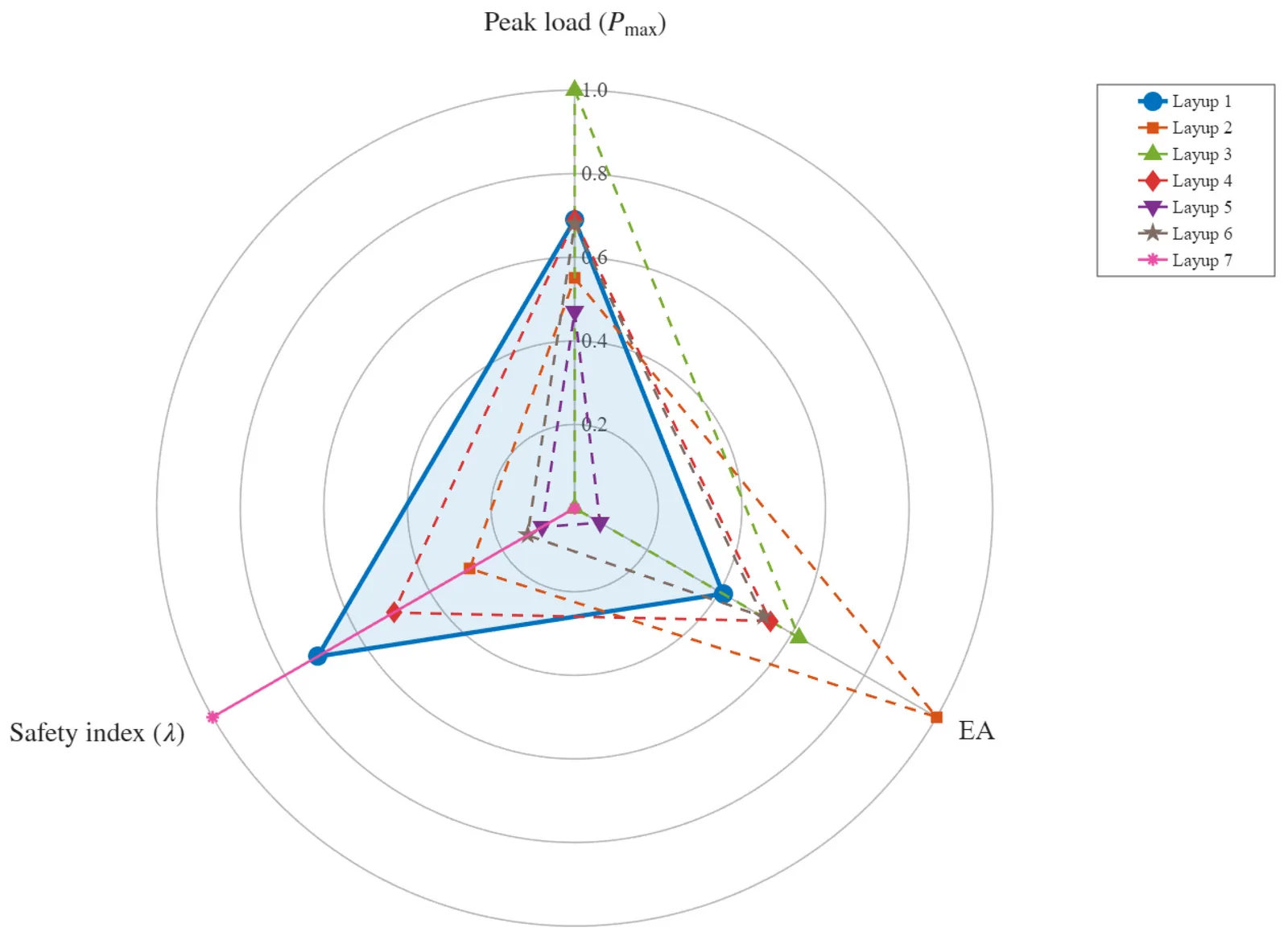
Radar representation of normalized performance for different laminate configurations.

The normalized indicators and CEI values are compared in Figure 11 and Figure 12. Marked differences were obtained in peak load, effective energy absorption, and failure safety. Several layups showed high performance in a single metric but remained unbalanced in the overall evaluation, confirming the limitation of single-indicator selection.

Layup 3 obtained the highest normalized score in peak load, consistent with the 28.38 kN maximum load reported in Section 3.1. Its failure-safety score, however, decreased to zero because of the abrupt brittle response identified in Section 3.2. The resulting comprehensive evaluation index was limited to 0.535. Layup 2 showed a relatively favorable energy-absorption performance, but its overall indicator distribution remained unbalanced. These results show that superiority in either maximum strength or energy absorption alone did not produce the highest comprehensive performance.

A substantially lower comprehensive score was obtained for Layup 5. After the post-splitting frictional contribution was excluded from the effective energy absorption according to the criterion defined in Section 2.4, the normalized performance of Layup 5 remained low and its CEI was 0.213. The result is consistent with the loss of hoop restraint and longitudinal splitting described in Section 3.2. Layup 1 showed a more balanced distribution among peak load, effective energy absorption, and failure safety. Its radar envelope was broader and more uniform than those of the other configurations, and the corresponding CEI reached 0.612, the highest value among the seven layups.

**Figure 13:**
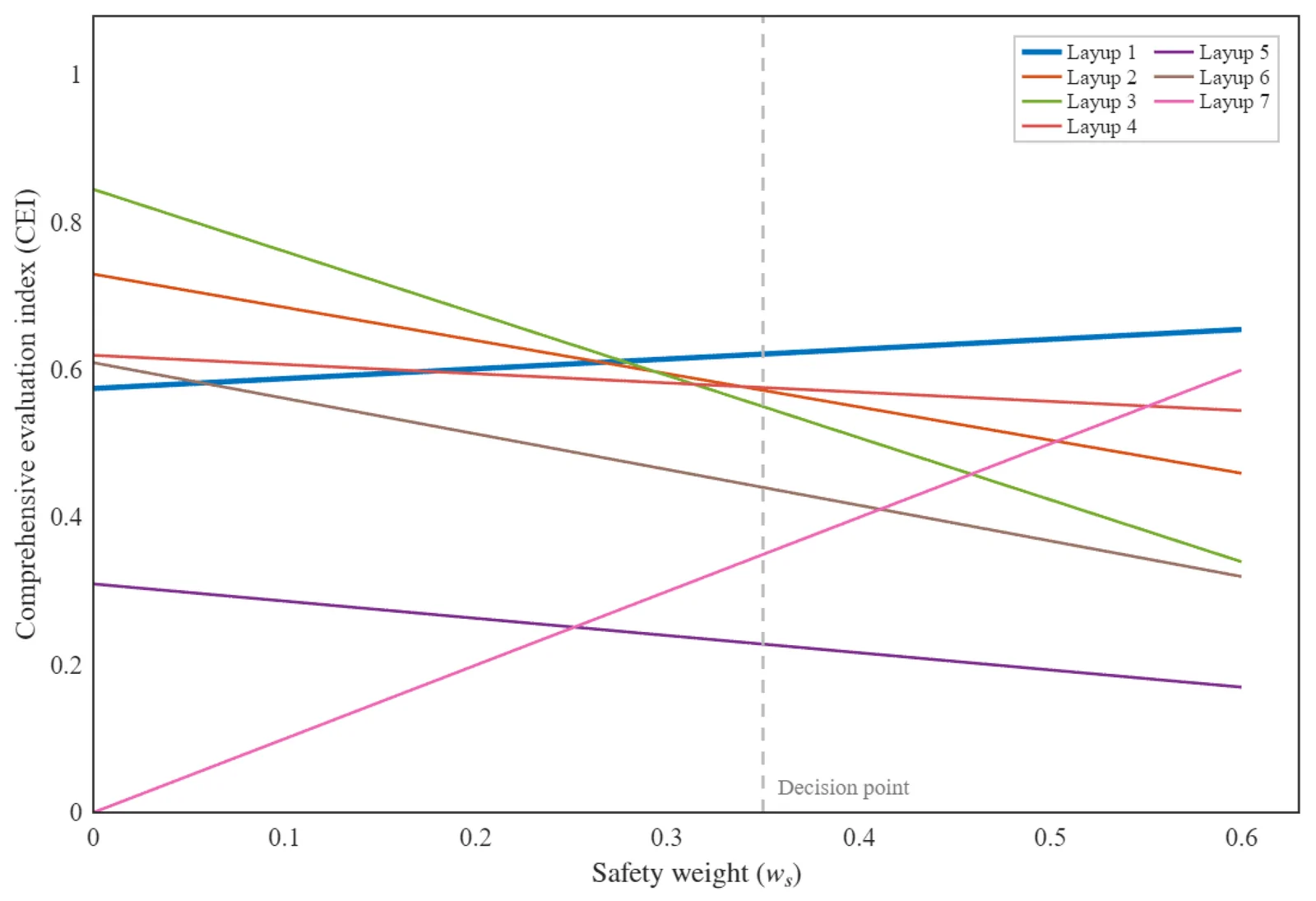
Sensitivity of CEI to variation in the failure-safety weight.

The sensitivity of the laminate evaluation to the safety weight is shown in Figure 13. When the safety weight remained below approximately 0.2, Layup 3 obtained a higher CEI because of its strength-dominated response. The CEI curves of Layups 1 and 3 intersected at approximately *w_s_* = 0.2. With a further increase in the safety weight, the CEI of Layup 3 decreased rapidly, whereas that of Layup 1 remained comparatively stable. The similar trend sensitivities of Layups 1 and 4 can be explained by their normalized constituent values in Figure 11. Both configurations have nearly identical normalized peak-load values, while Layup 1 has a higher safety index but lower effective energy absorption than Layup 4. These compensating differences result in comparable CEI variations as the safety weight changes. Overall, the sensitivity analysis indicates that the evaluation outcome depends on the relative emphasis placed on strength, energy absorption, and failure safety.

Among the seven investigated configurations, Layup 1 exhibited the most balanced numerical response in terms of load capacity, energy dissipation, and failure safety. This preference is restricted to the present material parameters, boundary conditions, and evaluation criteria and should not be interpreted as a generally optimal laminate configuration.

## 4. Conclusions

Seven laminate architectures were compared under pin-loaded axial compression. Layup 3 reached the highest peak load of 28.38 kN but exhibited abrupt brittle failure, whereas Layup 1 reached 22.02 kN with gradual post-peak degradation and localized progressive damage. Layup 7 showed the lowest peak load of 8.02 kN. Removal of hoop-oriented plies promoted longitudinal splitting, while asymmetric stacking reduced deformation stability.

A screening procedure combining progressive damage analysis with multi-criteria evaluation was established. Peak load, effective energy absorption, and failure safety were integrated into the CEI. Layup 1 achieved the highest CEI of 0.612, exceeding Layup 3 (0.535) and Layup 5 (0.213), and remained preferred when the safety weight exceeded approximately 0.2.

The proposed framework provides a numerical basis for preliminary laminate screening by considering load capacity, damage tolerance, energy dissipation, and deformation stability simultaneously. From a manufacturing perspective, Layup 1 employs conventional 0°, ±45°, and 90° ply orientations and is therefore compatible in principle with common CFRP tubular manufacturing routes. However, practical performance may be affected by ply angle deviation, ply thickness variation, hole-making damage, pin–hole fit tolerances, and variations in the composite–metal friction condition, which were not explicitly examined in the present numerical model. Experimental validation has not yet been performed; therefore, the reported laminate preference should be interpreted within the present numerical framework and the seven investigated configurations. Future work will focus on experimental validation and manufacturing-tolerance assessment.

## Figures and Tables

**Table 1:** Mechanical properties of the T300 CFRP laminate.

Elastic modulus (MPa)	*E*_11_	*E*_22_	*E*_33_
	114,000	8280	8280
Poisson’s ratio	*v*_12_	*v*_13_	*v*_23_
	0.31	0.31	0.40
Tensile strength (MPa)	*X_T_*	*Y_T_*	*Z_T_*
	1809.08	75.3	75.3
Shear modulus (MPa)	*G*_12_	*G*_13_	*G*_23_
	4280	4280	2852
Shear strength (MPa)	*S*_12_	*S*_13_	*S*_23_
	134.6	90	90
Compressive strength (MPa)	*X_C_*	*Y_C_*	*Z_C_*
	1163.8	183.88	183.88

**Table 2:** Intralaminar fracture energy in the Hashin model.

Damage Mode	Symbol	Fracture Energy (N/mm)
Fiber tension	*G_ft_*	80
Fiber compression	*G_fc_*	80
Matrix tension	*G_mt_*	1.0
Matrix compression	*G_mc_*	1.0

**Table 3:** Laminate configurations considered in the numerical analysis.

Layup	Stacking Sequence	Design Variable	Mechanical/Structural Characteristic
Layup 1	${\left[0^{\circ }/45^{\circ }/-45^{\circ }/90^{\circ }\right]}_{s}$	Baseline configuration	Balanced performance
Layup 2	${\left[0^{\circ }/30^{\circ }/-30^{\circ }/90^{\circ }\right]}_{s}$	Helix angle	Axial stiffness
Layup 3	${\left[0^{\circ }/60^{\circ }/-60^{\circ }/90^{\circ }\right]}_{s}$	Helix angle	Hoop stiffness
Layup 4	${\left[45^{\circ }/-45^{\circ }/0^{\circ }/90^{\circ }\right]}_{s}$	Stacking order	Buckling suppression
Layup 5	${\left[0^{\circ }/45^{\circ }/-45^{\circ }/0^{\circ }\right]}_{s}$	Hoop restraint	Longitudinal splitting
Layup 6	[0^∘^/45^∘^/−45^∘^/90^∘^/0^∘^/45^∘^/−45^∘^/90^∘^]	Stacking symmetry	Extension–bending coupling
Layup 7	${\left[\pm 45^{\circ }\right]}_{4}$	Axial load-bearing skeleton	Load-carrying limit

**Table 4:** Mesh-sensitivity analysis for the baseline Layup 1.

Mesh	Size (mm), Hole/Far	Elements	*P*_max_ (kN) [Δ%]	*EA_eff_* (J) [Δ%]
Coarse	0.75/3.0	7096	21.61 [2.22]	42.80 [3.82]
Medium	0.50/2.0	7327	22.02 [0.36]	44.10 [0.90]
Fine	0.35/1.4	7831	22.10 [0.00]	44.50 [0.00]

Note: Sizes are given as hole-edge/far-field values. Bracketed values denote relative differences with respect to the fine mesh.

## Data Availability

The original contributions presented in this study are included in the article. Further inquiries can be directed to the corresponding author.

## References

[1] Searle J., Meng M., Summerscales J. (2022). FEA modelling and environmental assessment of a thin-walled composite drive shaft. Thin-Walled Struct..

[2] Bilalis E.P., Keramidis M.S., Tsouvalis N.G. (2022). Structural design optimization of composite materials drive shafts. Mar. Struct..

[3] Qi L., Li C., Yu X., Min W., Shi H., Tao L., Wang H., Yu M., Ni L., Sun Z. (2021). Effect of reinforced fibers on the vibration characteristics of fibers reinforced composite shaft tubes with metal flanges. Compos. Struct..

[4] Jiao Y., Li Y., Huan D., Liu H., Luo R., Wang J. (2022). Forming and layering method optimization of prestressed joint of composite-metal drive shaft. J. Reinf. Plast. Compos..

[5] Ding G., Hou S. (2024). CFRP drive shaft damage identification and localization based on FBG sensing network and GWO-BP neural networks. Opt. Fiber Technol..

[6] Yang X., Liu X., Wang J. (2023). Random fatigue life analysis of carbon fiber-reinforced plastic for automotive drive shaft with environment temperature data. Fatigue Fract. Eng. Mater. Struct..

[7] Wang J., Qin T., Mekala N.R., Li Y., Heidari-Rarani M., Schröder K.-U. (2022). Three-dimensional progressive damage and failure analysis of double-lap composite bolted joints under quasi-static tensile loading. Compos. Struct..

[8] Cao Y., Zhi J., Zuo D., Li X., Cao Z., Wang Y., Tay T.E. (2023). Mesoscale modelling of progressive damage and failure in single-lap and double-lap thin-ply laminated composite bolted joints. Compos. Struct..

[9] Lin Q., Zhao Y., Pan W., Liu Y. (2023). An improved 3D model of composite bolted joints with detailed thread structure and progressive damage analysis of realistic tightening process. Compos. Struct..

[10] Zhuang W., Zhang H., Wang E., Chen S., Liu Y. (2023). A progressive damage model for carbon fiber-reinforced polymer laminates subjected to fastener pull-through failure. Compos. Struct..

[11] Lin Q., Zhao Y., Chen Y., Liu Y., Pan W. (2024). Progressive bearing failure analysis and strength prediction method for the initial assembly and tensile process of composite bolted joints. Compos. Part A Appl. Sci. Manuf..

[12] Wang S., Liu X., Huang B., Liao H. (2023). Fatigue life prediction of composite suspension considering residual stress and crack propagation. Proc. Inst. Mech. Eng. Part D J. Automob. Eng..

[13] Jiang L., Xiao S., Dong D., Yang B., Chen D., Yang G., Zhu T., Wang M. (2022). Experimental study of bonded, bolted, and hybrid braided CFRP joints with different stacking sequences and lapping patterns. Thin-Walled Struct..

[14] Wu Z., Li J., Shi L., Chen X. (2023). Effect of stacking configuration on the mechanical property and damage behavior of braided composite tube under three-point bending. Thin-Walled Struct..

[15] Zhang F., Lin Y., Zhang Z., Song C., Wang M. (2023). Effects of stacking sequences on the axial compressive buckling behavior and failure characteristics of channel-section CFRP/Al laminate columns. Compos. Struct..

[16] Lei Z., Pan R., Sun W., Dong Y., Wan Y., Yin B. (2024). Fatigue damage mechanisms and evolution of residual tensile strength in CFRP composites: Stacking sequence effect. Compos. Struct..

[17] Shi X., Liu X., Zhu M. (2023). Fatigue life prediction of eccentric springs for the automobiles considering residual stress. Fatigue Fract. Eng. Mater. Struct..

[18] Kapidžić Z., Ávila Granados D.L., Moreno Arias J.A., Quiroga Aguilera M.J., Casas Rodríguez J.P., García Callejas J.C. (2022). Bolt fatigue in CFRP joints. Int. J. Fatigue.

[19] Gamdani F., Boukhili R., Vadean A. (2022). Fatigue behavior of hybrid multi-bolted-bonded single-lap joints in woven composite plates. Int. J. Fatigue.

[20] Park S.M., Jeon J.H., Choi W.J. (2024). Study on bearing strength and failure modes of single bolted joint carbon/epoxy composite materials. Polymers.

[21] Qu H., Li D., Li R., Ge E., Zhai Y. (2023). Effect of forced assembly on bearing performance of single-lap, countersunk composite bolted joints—Part II: Numerical investigation. Compos. Struct..

[22] Zhang X., Liu X., Wang S. (2025). Random fatigue life prediction of composite components for double-wishbone suspension considering an improved crack propagation model. Proc. Inst. Mech. Eng. Part D J. Automob. Eng..

[23] Qu H., Li D., Zhai Y., Ge E., Xi W., Ji C. (2024). Experimental investigation on the effect of forced assembly on fatigue behavior of single-lap, countersunk composite bolted joints. Int. J. Fatigue.

[24] Qin Z., He Y., Wang S., Meng C. (2023). Investigation of CFRP-countersunk bolted assembly fatigue damage under three-point bending via experimental and numerical analysis. Polymers.

[25] Zou F., Dang J., Chen T., An Q., Chen M. (2023). Evaluation of typical hole-making strategies on mechanical behavior of CFRP/Ti single-lap bolted joints. Compos. Struct..

[26] Feng J., Zhang J., Jin W., Liao R. (2024). Damage evolution modeling of CFRP/Al single-lap screw type blind riveted joints during realistic installation process. Compos. Struct..

[27] Cao Y., Zuo D., Zhao Y., Cao Z., Zhi J., Zheng G., Tay T.E. (2021). Experimental investigation on bearing behavior and failure mechanism of double-lap thin-ply composite bolted joints. Compos. Struct..

[28] Erbil E., Karakuzu R. (2024). Fastening solutions on composite structures: Progressive damage analysis of a unique bolted joint design for single-lap CFRP laminate. Mech. Adv. Mater. Struct..

[29] El-Sisi A.E.-D.A., Salim H.A., Gomaa S.A., El-Feky M.H. (2025). Progressive failure analysis of woven and non-woven CFRP composite/steel bolted joints. Polym. Compos..

[30] Jiang C., Liu X., Zhang M., Wang X., Wang Y. (2021). An improved nonlinear cumulative damage model for strength degradation considering loading sequence. Int. J. Damage Mech..

[31] Zhang W., Wang J., Zhou J., Guo Y., Zhang D., Duan Y., Guan Z., Cantwell W.J. (2025). Damage progression and failure in L-shaped laminated composite bolted joints subjected to quasi-static tensile loading. Compos. Struct..

[32] Liu X., Huang K., Han X., Zhou J., Zhang L., Guo L. (2025). Investigation of temperature and structural configuration effects on the mechanical properties of CFRP bolted joints. Compos. Struct..

[33] Liu X., Huang K., Han X., Liang D., Wang X., Zhang L., Guo L. (2025). Temperature effect on the fatigue properties of CFRP bolted joints by combining DIC, AE and IRT techniques. Thin-Walled Struct..

[34] Lin Q., Zhao Y., Liu Y., Pan W., Ren Y., Yu W. (2025). Optimization of assembly parameters for composite bolted joints aiming at time-varying bearing reliability improvement. Compos. Struct..

[35] Chen L., Lin J., Li M., Xu Q., Zhu W., Ke Y. (2026). Multiscale modeling for bearing performance and progressive failure analysis of interference-fit CFRP joints. Compos. Struct..

[36] Zhai Y., Qu H., Li D., Ge E., Xiao R., Yang J. (2025). Effect of bolt preload uncertainty on fatigue reliability of single-lap, countersunk composite bolted joints considering forced assembly interaction. Int. J. Fatigue.

[37] Wang L., Fan Y., Hu W., Li Y., Zhang K., Cheng H., Bai R. (2026). Effect of installation and preloading on tensile failure mechanism of composite countersunk sleeved interference-fit joints. Thin-Walled Struct..

[38] Liu C., Kong X., Zhou Q. (2026). A new threaded insert reinforced joint to achieve ultra-high performance of CFRP bolted connections. Compos. Part B Eng..

[39] Gerendt C., Hematipour M., Englisch N., Scheffler S., Rolfes R. (2023). A finite element-based continuum damage model for mechanical joints in fiber metal laminates under static and fatigue loading. Compos. Struct..

[40] Zhai Y., Qu H., Li R., Ge E., Li D. (2023). Effect of forced assembly on bearing performance of single-lap, countersunk composite bolted joints—Part I: Experimental investigation. Compos. Struct..

[41] Zhang M., Zhang Y., Zhang Z., Cui X., Fu K., Li Y. (2026). Effects of process parameters on anisotropic mechanical properties of 3D printed short carbon fiber-reinforced polyamide composites. Polym. Compos..

[42] Yang B., Li A., Wu J., Qaiser Z., Fu K., Yang D. (2026). Strain-rate dependence of impact behaviour in carbon fibre composites. Thin-Walled Struct..

[43] Taimour M., Abd-Elhady A.A., Sallam H.E.-D.M., Sayed S.A.A. (2023). Implementing functionally graded fibers technique to enhance pinned-joint performance in cross-ply laminate polymeric composites. Compos. Struct..

[44] Wang S., Wang D. (2020). Optimization design of carbon fiber reinforced polymer anti-collision beam crashworthiness by grey relational analysis with entropy method. Acta Mater. Compos. Sin..

[45] Wang X., Wang Y., Ji Y., Hu H., Cao D., Zheng K., Liu H., Li S. (2022). Modeling progressive damage and failure of single-lap thin-ply-laminated composite-bolted joint using LaRC failure criterion. Materials.

[46] Koord J., Stüven J.-L., Völkerink O., Petersen E., Hühne C. (2022). Investigation of exact analytical solutions for composite laminates under pin-bearing loading. Compos. Struct..

[47] Li X., Kumar S., Hwang D.-W., Shin D.-H., Kim Y.-H. (2023). Pin-loaded tensile behavior and failure analysis of CF/PEKK composites under extreme temperature. Compos. Part A Appl. Sci. Manuf..

[48] Liu G., Li C., Luo W., Liao F., Zhang Y., Zeng S. (2023). Investigation on the progressive damage and bearing failure behavior of composite laminated bolted joints under tension. Crystals.

